# Coinciding revolutions: how discovery of non-coding DNA and RNA can change our understanding of addiction

**DOI:** 10.3389/fgene.2012.00271

**Published:** 2012-11-23

**Authors:** Andrzej Z. Pietrzykowski

**Affiliations:** Laboratory of Adaptation, Reward, and Addiction, Department of Animal Sciences, Department of Genetics, Rutgers University, New Brunswick, NJ, USA

*“Omnia mutantur, nihil interit”* (Everything changes, nothing perishes)

–Ovid, Metamorphoses, AD 8

The twenty-first century started with the Big Bang—the first ever sequencing of the human genome in 2000/2003 (Lander et al., 2001; Venter, 2003), and since then our scientific Universe started to expand with the speed of light. This year (2012) the Supernova exploded—the Encode project, a series of thirty papers, trashed the “junk DNA” concept. The project indicates that the vast majority (99%) of the human genome, although non-coding, is not “junk” but is biologically active and essential to maintain fundamental processes in a cell (Bernstein et al., 2012). One of the earlier stellar breakthroughs was the discovery of microRNA, a short RNA species crucial for regulation of gene expression (Lee et al., 1993). With discoveries like these, it becomes more and more obvious that our biological Universe is much more complex and dynamic than we have ever imagined. It is very probable that many biological rules and regulations still await discovery. This is particularly important for our understanding of the mechanisms underlying the development of chronic diseases, like addiction. Addiction is a persistent, neurobiological disease affecting primarily the brain's *reward system*, which consists of a circuitry of several interconnecting brain regions, including the prefrontal cortex, nucleus accumbens, ventral tegmental area, amygdala, paraventricular nucleus, and others (Koob and Le Moal, 2001). The reward system is one of the most important physiological features of the brain. We use it constantly, unconsciously, to make countless decisions essential to complete simple and complex tasks, by performing risk/benefit ratio analyses. This powerful motivator drives human and animal behavior, allows an individual to thrive and ultimately, for a species to propagate.

Some drugs target the reward system and can drastically change the behavior of individuals vulnerable to *addiction*, which is characterized as the refocusing of all activities on one goal—the continued intake of the drug. The drug's effect on the CNS is so powerful that the individual will continue to use it despite any associated adverse consequences. Neurobiological models of addiction try to explain how chronic or frequent exposure to addictive drugs hijacks the elements of the brain reward circuitry and causes transition from voluntary drug-taking to habitual and compulsive drug-seeking (Hyman and Malenka, 2001). During this transition, neuronal *adaptations* occur, which perpetuate and intensify the disease. Escalating drug intake due to *tolerance* causes incessant transition of the allostatic set-point, which fails to maintain system stability. Usually, drug addiction and alcohol addiction are described separately, but at the biological level both addictions have common core changes in the reward system through similar neuronal adaptations. However, in either type of addiction, many molecular underpinnings are still unknown.

Recently, several papers described the involvement of different non-coding RNAs, primarily microRNA, in unraveling novel molecular mechanisms of this debilitating disease. We put together this Special Issue of Frontiers in Genetics to provide our readers with a comprehensive overview of current research on non-coding RNA in both drug and alcohol addiction. We invited leaders in this dynamic new field to write a series of reviews, and other prominent scientists to share their views of this subject through a series of editorials. This special issue is divided into five chapters: alcohol, nicotine, cocaine, morphine, and bioinformatics.

*Alcohol* is a simple product of yeast fermentation, and is fairly easy to obtain. People have been using alcohol probably from the beginning of recorded history. Alcohol addiction (alcoholism) is the oldest and most widespread type of addiction. In this special issue, Nunez and Mayfield (2012) describe microRNA signatures in the brains of alcoholics and the process of microRNA modulation of epigenetic nuclear mechanisms. Miranda (2012) discusses teratological consequences of microRNA disregulation by alcohol during fetal development. The editorial by Reilly (2012) explains how studying microRNA can provide new perspectives and treatments of alcoholism and Fetal Alcohol Spectrum Disorder (FASD).

*Nicotine* is primarily inhaled during the smoking of dried tobacco leaves (*Nicotiana tabacum*), but also can be consumed in other forms. Nicotine addiction has spread quickly around the world in the post-Columbian era, becoming a major, present-day health issue in many countries. According to Maccani and Knopik (2012), detrimental effects of nicotine start very early—*in utero*. They describe in their review how maternal cigarette smoking during pregnancy alters expression of selected microRNAs in placenta. They also discuss the effect of cigarette smoking on microRNA and long non-coding RNA (lncRNA) expression in the epithelium of airways, which is important in the pathogenesis of cancer. Their review indicates that environmentally regulated epigenetic changes affect health throughout the course of one's life, while an editorial by Ehringer (2012) describes the presence of “critical periods” during which the environmental effect is particularly strong.

*Morphine* is the most abundant alkaloid found in opium (opiate), a product of the poppy fruit (*Papaver somniferum*). Morphine has been used for centuries to treat pain. However, morphine and related products have very potent addictive properties. Currently, addiction to morphine and related drugs is rising, mainly in affluent countries. Several microRNAs have been shown to be important in morphine actions. In this Special Issue, Rodriquez (2012) summarizes the regulation of miR-133b by morphine and its possible involvement in morphine addiction and cancer. He and Wang (2012) describe the role of let-7, the first identified human microRNA, in regulation of morphine receptors important during the development of opioid tolerance. Zheng et al. (2012) center their review on microRNA-23b and -190. They also propose that a non-coding RNA relevant to opioid addiction can belong to one of two groups: (1) directly regulated by opioids, or (2) not-regulated by opioids but controlling pathways leading to addiction. Wood and Lipovich (2012), in their editorial, stress the importance of a systematical examination of the evolutionary conservation of microRNAs and their targets in opioid research.

*Cocaine* is an alkaloid obtained from the leaves of the coca plant (*Erythroxylum* family). Chewing raw coca leaves seems to be non-addictive, probably because of a very small content (<1%) of cocaine in the leaves. Coca in this form is a mild stimulant and analgesic and also a source of nutrients. However, purified cocaine has strong addictive properties. Currently, cocaine addiction is on a rise, and cocaine addicts can be found in many corners of the world. Wu (2012) in his editorial emphasizes advantages of a novel approach to study cocaine addiction using next-generation sequencing. The powerful results of this great methodology are appreciated by the review by Eipper-Mains et al. (2012). The review by Bali and Kenny (2012) describes their elegant studies implicating the regulation of CREB, MeCP2, and BDNF pathways by specific microRNAs (miR-132 and -212) in the behavioral response to cocaine.

We added a special chapter on *bioinformatics* because wet lab experimental approaches to microRNA research are initiated or complemented by bioinformatical experiments. There are several challenges to ensure synergism between next-generation techniques and bioinformatic analyses as emphasized in the editorial by Liu et al. (2012). The review by Mu and Zhang (2012) provides a comprehensive comparison of several bioinformatical resources important in addiction research. This special chapter also contains an article by Sartor et al. (2012), which draws our attention not only to the importance of microRNA but also to its “longer” brother—the lncRNA, in persistent neuronal maladaptation associated with addictive behaviors.

Research trying to understand involvement of various types of non-coding RNA in the development of addictions is at its early stages. Not all of the addictive substances have been yet screened. Moreover, an addiction can occur not only to exogenous chemicals but also to other cues like gambling, food, internet etc. Future research should look into the misregulation of gene expression by non-coding RNA in all the different types of addiction (Pietrzykowski, 2010). Nevertheless, the work discussed in this Special Issue has already left a strong legacy: there is no doubt that non-coding RNAs play absolutely essential roles in processes controlled by addictive drugs. Thus, to truly understand addictions, one must study non-coding RNA.

Research in non-coding RNA will probably increase exponentially, because we are now at a really advantageous situation in biological history—an intersection of several revolutions. *The technical revolution* provides the increasing ability to perform a deep, accurate, and fast sequencing of entire genomes. *The bioinformatical revolution* provides more and more sophisticated tools to analyze gigabytes of information delivered by sequencing. *The genome structure revolution* has started to disentangle intertwined genomic three-dimensional networks of interactions between genes and gene regulatory regions. Inevitably, we have started to experience *the conceptual revolution*: we realize that the regulation of gene expression is a multilevel interactive process, much more complex than we initially thought, and that messenger RNA can regulate each other via miRNA sequestering as proposed in the ceRNA (complementary endogenous) theory (Salmena et al., 2011), and that circulating exosomes can contain gene regulatory material, like microRNA and lncRNA, enabling a brand new cell-to-cell communication route (Valadi et al., 2007). Hopefully, these coinciding revolutions will ultimately provide fundamental breakthroughs and create radical new treatment approaches to addiction disorders, benefiting the entire society.

We hope you will find this Special issue of Frontiers in Genetics truly engaging and share our enthusiasm that we are living in one of the most exciting time in scientific history, on a cusp of a cosmic scale of discovery in biological sciences.
